# Supplementary material for: Infective prey leads to a partial role reversal in a predator-prey interaction
Source: PLoS One. 2021 Sep 17;16(9):e0249156. doi: 10.1371/journal.pone.0249156 (PMC8448379; doi:10.1371/journal.pone.0249156)
Supplement: Attachment — Submitted filename: Review.docx [file pone.0249156.s001.docx]

**Review**

This study considers how role reversal in a predator-prey system may affect the persistence of the prey and the predator species. Although the focus of the study and thus the ecological question is very interesting, I am not entirely convinced that the current formulation of the model may significantly enlarge our understanding of the potential importance of role reversal within natural predator-prey systems (for details see below).

Hence, in my opinion, major revisions are required before this article is ready for publication.

**Major**

***Novelty and Reliability of results***

The two main results are not really surprising. First of all, it is almost immediately clear that the way in which the partial role reversal of the predator-prey interaction is modelled is stabilizing the corresponding population dynamics because it mainly acts to weaken the original predator-prey interaction. Furthermore, it is well known that a predator-prey model with a logistically growing prey and a predator with a type I functional response gives rise to a stable interior equilibrium assuming its existence.

At first glance, I was also quite confused by the result that the prey may even persist when its intrinsic growth rate is zero (Lines 343-348) purely from an energetic point of view since energy is irrefutable lost at each instance of feeding. Although the model may actually give rise to such an outcome, it entirely relies on the fact that the predator can allocate additional energy from an external pool which in the end is partly accessible to the prey. Is this really something we can expect in natural systems to happen?

Although, I guess, the results of when to expect predator or prey extinction or coexistence of both species have some value, I was still wondering whether the model is really able to describe the empirical system used for parameterization and to motivate the study in the first place, given the strong phenomenological nature of the model (for further comments on the model see below). I mean, the authors state that their model may also describe a system where a large number of sticklebacks may feed on young pikes and thus potentially vulnerable individuals of their predators by isolating these weak individuals (lines 415-419), I that the feeding interaction between the two fish species is also not well described by the model presented in this study. In fact, the model presented here assumes that the prey is first consumed by the predator before it is going to utilize the corresponding predator individuals. In contrast, in the predator-prey system that comprises sticklebacks and pikes, the prey is not going to be eaten by the predator before the predator gets vulnerable to predation by the prey. To analyze such a predator-prey system with partial role-reversal, the sole ingredient that needs to be accounted into a model is stage structure within the predator population, where the juvenile state is vulnerable to predation by the prey of the adults.

***Model formulation***

The phenomenological model presented describes a potential mechanism that may lead to the reversal of the predator-prey interaction in a very simplistic way that makes it difficult to actually identify the ecological processes that may act in the first place. For example, the bacterial prey is assumed to be partly able to infect and subsequently to feed and grow on the susceptible predator. However, given the huge size differences of the pathogen and host species considered, part of the population growth of the bacteria should actually taking place within the host. Furthermore, I would generally expect that an infectious bacterium would need some time to grow within the host before the host is actually getting sick, which would shift sick individuals from the susceptible to the infected stage.

The model also makes some other strong assumptions that are not well justified. For example, what is the reason behind the assumption that the infected predators cannot feed any longer on the prey? Why did you assume a functional type I response and not a more realistic functional type II response? When both species are assumed to be generalists, I was wondering why the authors entirely excluded the possibility for cross-feeding and thus interspecific competition. I mean, the bacteria may also infect and consequently feed and grow on some other species that is also a prey of the predator. Most strikingly, the model assumes that the bacterial trait of being (non)infectious is not heritable. In fact, being infectious or non-infectious is treated more like a stochastic process with alpha denoting the average probability of a bacteria cell being infectious or not. Is there any empirical evidence that supports this assumption?

Based on the model formulation (lines 96-100), the attack rate of the predator should have the unit 1/(time*density) (cf. Table 1). This contrasts with the unit that is obtained at a later step where the attack rate is further specified through other parameters. In particular the odd scaling with the volume of an artificial feeding unit leads to a wrong dimension of the attack rate and potentially also wrong estimate of the attack rate. Hence, please correct this mistake so that the units of the parameters and state variables match up. This implies that the model parameterization and corresponding simulation results need to be revised. Furthermore, I was wondering why the attack rate needs to be restricted to the finite interval [0,1] (line 104)? I don’t see any clear reason for this choice.

***Presentation of results***

Overall, the presentation of the results lacks a clear structure and wording, contains redundant information and uses misleading terminology.

For example, the Method section partly contains results (Lines 198-217). In addition, some results are described twice, e.g. text in lines 248-250 restate results from lines 219-221, text in lines 230-234 cover the content of lines 256-259, results described in lines 287-288 are partly restated in lines 301-302 and the sentence from lines 275-276 restates results from lines 230-235 again. Please revise the text in order to improve readability.

It also remains unclear to me why the intrinsic growth rate r of the prey that is associated with the logistic growth terms are sometimes called environmental growth rate (e.g. lines 219ff) or outside prey growth rate (e.g. line 230) despite being introduced simply as the growth rate of the infective prey (Table 1). Please use clearly defined terminology throughout the manuscript.

The details about the nature of the fixed points and their stability and how the fixed points are calculated (cf. 161 - 188) and how their stability is determined (cf. lines 306 – 368) should go into an Appendix as it only supports numerically derived simulation results but does not seem to add further insights here. Based on this I also think that the presentation of the simulation and analytical results should be combined as they do only support but not complement each other.

Different aspects of the model results are confusingly described in the paragraph at lines 230-242. On the one hand, the authors try to make the point that the impact of an increased intrinsic growth rate on the prey biomass depends on the actual infectivity of the prey. On the other hand, an increase in the infectivity of the prey also influences the prey and predator abundances itself. The presentation of these results is far from being clear. Furthermore, the main text comprises a lot of information that belongs to the Figure legends (e.g. lines 237ff, 282ff, 302ff).

**Minor**

**Table 1:** Why calling eSI a conversion rate? It seems rather to be an infection/infectivity rate.

Line 35: What do you mean by “normal growth rate”? Please clarify

Line 47: Did you mean here “survive” instead of “coexist”? Please clarify.

Line 49: The position of the reference “[5]” seems to be misplaced.

Line 50: It should read predator***-prey*** interaction. Please revise.

Line 54: Please omit “in the community” because it suggests that you have modelled many more species.

Line 60: Delete the “.” in front of the reference.

Line 60: It should read “According ***to***”. Please revise.

Lines 62-65: Reads very general. Can you be more specific about your findings?

Lines 80-83: Please, delete this paragraph as it is totally redundant to the text provided in lines 68-76.

Lines 103: Please add “in the absence of the other species”.

Line 107: The total number of prey individuals harvested is given by a*C****S*** and not a*C!

Line 119: Please delete “of the” form the sentence as it is written down twice.

Lines 122/123: This sentence reads odd. Please clarify.

Lines 207/208: Please delete this sentence as it is part of the methods.

Lines 219, 221, 223, 225 & others: Why is the intrinsic growth rate called an environmental growth rate? Please clarify or revise.

Line 219: What do you mean with an opportunistic prey? Please clarify.

Line 244-246: This sentence does not add any explanation but restates the observation twice. Please revise.

Line 252/253: What do you mean with the order of the population sizes?

Line 254: Delete “to” from the sentence.

Line 261: Please add a “to” in front of “a”.

Lines 273/274: Please delete Fig.3 in front of the subheading.
