# Supplementary material for: Infective prey leads to a partial role reversal in a predator-prey interaction
Source: PLoS One. 2021 Sep 17;16(9):e0249156. doi: 10.1371/journal.pone.0249156 (PMC8448379; doi:10.1371/journal.pone.0249156)
Supplement: Attachment — Submitted filename: Responses to Rewievers.docx [file pone.0249156.s003.docx]

**Our answers to the review comments have been organized as follow:**

- **Editor**
- **Referee 1**
- **Referee 2**

Comments by the Editor

I encourage you to investigate infectious disease models (both SIR models and vector-born disease models). The presented model looks like that of a vector-born disease with a part of prey is infected with a pathogen.

**Answer:** *We do not see how our model would fit into the scope of SIR molels. We continue defending our decision to consider the species interaction as a case of partial role reversal in a predator prey system.*

*In SIR models the basic mechanism is contact infection by infected individuals, which we do not have. SIR, usually in the form of compartments, is a single species model whereas our model is based on a predator-prey model. The infection is due to the second species. The infection occurs through grazing, that is, killing the individuals of another species.*

*We do not see in our model any component which could be interpreted as a “vector”. The infective bacteria it cannot be. Naming the sediment hosting the bacteria as a vector would stretch the definition of a vector so much that it would become useless.*

Additional Editor Comments:

As reviewer 1 points out, it is not clear how representative the model is. Furthermore, you cannot stabilize a system that is already "stable". Looking at the figures, I think the system is already stable under a wide range of parameter space (including alpha=0).

**Answer:** *Our wording is “stabilizes the predator-prey interaction” which in Lotka-Volterra case is unstable.*

You can have r_c=0 or positive as well as r_s=0 or positive, representing whether prey (or predator) is specialist or generalist. Analogous to mutualism models, we could also have k_c<0 or >0 as well as k_s<0 or >0, depending on whether they are obligate or facultative. I think investigating the dynamics of the model under each combinations of these conditions is potentially interesting (and some of them will produce “instability” without infection). -- just a suggestion for improvements.

**Answer:** *Thank you for the encouragement. We agree that the questions of specialist-generalist or mutualist species, and others, are very interesting. We also got a list of detailed questions of other modification from the two referees, on the top of our own list of questions that we have needed to leave out in order to keep the manuscript comprehensible.*

As reviewer 2 points out, the model is also like SIR models. A large body of literature on SIR models exist. The model results should also be discussed in terms of the existing knowledge of the SIR models. I personally think the model is more like a vector-born disease model rather than an SIR model. Right now, the model is interpreted as having prey that is an energy source as well as pathogenic. However, it is more natural to interpret that the prey is carrying a pathogen (e.g. virus, bacteria, trematode, nematode, myxozoan, etc.). It may be easier to justify the model as a vector-born disease. Similarly to SIR models, there are a large body of literature on vector-born diseases. I would not be surprised if someone else already developed the model like the one presented (e.g. malaria infection model).

**Answer:** *We commented this in our first answer above*.

Reviewer 2 also raised some concerns with regard to the units of parameters. Some justifications of the parameter estimations should be presented.

**Answer:** *We have revised the parameter section to clarify the presentation*.

**Reviewer 1**

Reviewer #1: Dear authors,
your study considers how role reversal in a predator-prey system may affect the persistence of the prey and the predator species. Although the focus of your study and thus the ecological question is very interesting, I am not entirely convinced that the current formulation of the model may significantly enlarge our understanding of the potential importance of role reversal within natural predator-prey systems (for further details see my complete review attached).
Sincerely yours

**Answer:** *Thank you very much for this view. I have carried out a major revision to improve the manuscript.*

**Review**

This study considers how role reversal in a predator-prey system may affect the persistence of the prey and the predator species. Although the focus of the study and thus the ecological question is very interesting, I am not entirely convinced that the current formulation of the model may significantly enlarge our understanding of the potential importance of role reversal within natural predator-prey systems (for details see below).

Hence, in my opinion, major revisions are required before this article is ready for publication.

**Major**

***Novelty and Reliability of results***

The two main results are not really surprising. First of all, it is almost immediately clear that the way in which the partial role reversal of the predator-prey interaction is modelled is stabilizing the corresponding population dynamics because it mainly acts to weaken the original predator-prey interaction. Furthermore, it is well known that a predator-prey model with a logistically growing prey and a predator with a type I functional response gives rise to a stable interior equilibrium assuming its existence.

**Answer:** We fully agree. However, adding one dimension to the model may change the stability properties. Thus, we felt that this result should be presented.

At first glance, I was also quite confused by the result that the prey may even persist when its intrinsic growth rate is zero (Lines 343-348) purely from an energetic point of view since energy is irrefutable lost at each instance of feeding. Although the model may actually give rise to such an outcome, it entirely relies on the fact that the predator can allocate additional energy from an external pool which in the end is partly accessible to the prey. Is this really something we can expect in natural systems to happen?

**Answer:** If the predator population size increases due to other resources it can be expected that it benefits the infective prey population.

Although, I guess, the results of when to expect predator or prey extinction or coexistence of both species have some value, I was still wondering whether the model is really able to describe the empirical system used for parameterization and to motivate the study in the first place, given the strong phenomenological nature of the model (for further comments on the model see below). I mean, the authors state that their model may also describe a system where a large number of sticklebacks may feed on young pikes and thus potentially vulnerable individuals of their predators by isolating these weak individuals (lines 415-419), I that the feeding interaction between the two fish species is also not well described by the model presented in this study. In fact, the model presented here assumes that the prey is first consumed by the predator before it is going to utilize the corresponding predator individuals. In contrast, in the predator-prey system that comprises sticklebacks and pikes, the prey is not going to be eaten by the predator before the predator gets vulnerable to predation by the prey. To analyze such a predator-prey system with partial role-reversal, the sole ingredient that needs to be accounted into a model is stage structure within the predator population, where the juvenile state is vulnerable to predation by the prey of the adults.

**Answer:** Obviously, we have failed in explaining the essence of partial role reversal. The pike-stickleback example seems to be misleading. We have now added the following clarifying material in the beginning of the introduction:

“Role reversal has been reported in cases, where the reversal coincides with age-depended size categories [3], age-depended susceptibility to predation [2], or changes in relative population abundances [1]. These role reversals are complete and include the idea that at some age or size a species switches being predated to being a predator itself. For example, the pike may be subject to predation by sticklebacks when it is small, but it develops into a real predator with increasing size.

However, if the role reversal is not complete the predator continues to hunt the prey while becoming vulnerable to the predation itself. In the partial role reversal the growth of the prey population relies on the prey's growth rate and on the additional resource acquiring by the infectivity, in particular, by its efficiency in killing and converting the predator into nutrition.“

***Model formulation***

The phenomenological model presented describes a potential mechanism that may lead to the reversal of the predator-prey interaction in a very simplistic way that makes it difficult to actually identify the ecological processes that may act in the first place. For example, the bacterial prey is assumed to be partly able to infect and subsequently to feed and grow on the susceptible predator. However, given the huge size differences of the pathogen and host species considered, part of the population growth of the bacteria should actually taking place within the host. Furthermore, I would generally expect that an infectious bacterium would need some time to grow within the host before the host is actually getting sick, which would shift sick individuals from the susceptible to the infected stage.

**Answer:** We are very well aware of these problems. We are lacking many details of the interaction between the sea cucumber and *V. splendidus*. We have included in our model the most important ones: the sea cucumber prays on the bacteria and *V. splendidus* may infect the sea cucumber. These facts supports the basic structure of the model of partial role reversal.

The model also makes some other strong assumptions that are not well justified. For example, what is the reason behind the assumption that the infected predators cannot feed any longer on the prey? Why did you assume a functional type I response and not a more realistic functional type II response? When both species are assumed to be generalists, I was wondering why the authors entirely excluded the possibility for cross-feeding and thus interspecific competition. I mean, the bacteria may also infect and consequently feed and grow on some other species that is also a prey of the predator. Most strikingly, the model assumes that the bacterial trait of being (non)infectious is not heritable. In fact, being infectious or non-infectious is treated more like a stochastic process with alpha denoting the average probability of a bacteria cell being infectious or not. Is there any empirical evidence that supports this assumption?

**Answer:** We are very well aware of this list of omitted ideas and details in the model. Ourselves, we have longer list. However, we feel that including more mechanisms in the model would not make presenting the idea of partial role reversal more clear. Some of the details would be insignificant (e.g., infected predators continuing feeding) or complicating the model and the analyses unnecessarily (e.g., heritability in the bacteria).

Based on the model formulation (lines 96-100), the attack rate of the predator should have the unit 1/(time*density) (cf. Table 1). This contrasts with the unit that is obtained at a later step where the attack rate is further specified through other parameters. In particular the odd scaling with the volume of an artificial feeding unit leads to a wrong dimension of the attack rate and potentially also wrong estimate of the attack rate. Hence, please correct this mistake so that the units of the parameters and state variables match up. This implies that the model parameterization and corresponding simulation results need to be revised. Furthermore, I was wondering why the attack rate needs to be restricted to the finite interval [0,1] (line 104)? I don’t see any clear reason for this choice.

**Answer:** We have rewritten the chapter “parametrization” to clarify the derivation of the parameter values and units.

We maintain our definition of the grazing rate (former “attack rate”): “can be interpreted either as a fraction of the feeding area grazed during a time step, or it can equally be interpreted as the prey selectivity coefficient of the predator“

***Presentation of results***

Overall, the presentation of the results lacks a clear structure and wording, contains redundant information and uses misleading terminology.

For example, the Method section partly contains results (Lines 198-217). In addition, some results are described twice, e.g. text in lines 248-250 restate results from lines 219-221, text in lines 230-234 cover the content of lines 256-259, results described in lines 287-288 are partly restated in lines 301-302 and the sentence from lines 275-276 restates results from lines 230-235 again. Please revise the text in order to improve readability.

**Answer:** Corrected. The results have been removed from the Method section s (Lines 198-217).

Repetitive sentence deleted from lines 248-250.

text in lines 230-234 cover…Corrected by shortening the figure legend. of Fig 3.

text in lines 287-288 and 301-302. Corrected by deleting the sentence on lines 3301-302.

Lines 275-76 ja 230-235 Corrected. Sentence on lines 275-276 is deleted.

It also remains unclear to me why the intrinsic growth rate r of the prey that is associated with the logistic growth terms are sometimes called environmental growth rate (e.g. lines 219ff) or outside prey growth rate (e.g. line 230) despite being introduced simply as the growth rate of the infective prey (Table 1). Please use clearly defined terminology throughout the manuscript.

**Answer:** Corrected

The details about the nature of the fixed points and their stability and how the fixed points are calculated (cf. 161 - 188) and how their stability is determined (cf. lines 306 – 368) should go into an Appendix as it only supports numerically derived simulation results but does not seem to add further insights here. Based on this I also think that the presentation of the simulation and analytical results should be combined as they do only support but not complement each other.

**Answer**: As the second referee did not express any wish to this direction and as we understand that there are different preferences on the availability of the analyses at hand while reading the article we decided not to move the material into an appendix. This is also affected by the fact that a new material related to the analysis of the switch point relies heavily on the equilibrium results.

Based on this we also think that the presentation of the simulation and analytical results should be combined as they do only support but not complement each other.

**Answer**: Corrected as suggested.

.

Different aspects of the model results are confusingly described in the paragraph at lines 230-242. On the one hand, the authors try to make the point that the impact of an increased intrinsic growth rate on the prey biomass depends on the actual infectivity of the prey. On the other hand, an increase in the infectivity of the prey also influences the prey and predator abundances itself. The presentation of these results is far from being clear. Furthermore, the main text comprises a lot of information that belongs to the Figure legends (e.g. lines 237ff, 282ff, 302ff).

**Answer**: General: figure legends are now improved to be detected more easily.

lines 237ff are figure legend. However, the figure legend has been shortened and the figure has been interpreted in the main text.

lines 282-282 are a part of the figure legend. The figure legend is now partly rewritten.

lines 302-305 are a part of the figure legend.

**Minor**

**Table 1:** Why calling eSI a conversion rate? It seems rather to be an infection/infectivity rate.

**ANSWER**: Corrected.

Line 35: What do you mean by “normal growth rate”? Please clarify

**ANSWER**: “normal” deleted.

Line 47: Did you mean here “survive” instead of “coexist”? Please clarify.

**ANSWER**: Corrected as suggested.

Line 49: The position of the reference “[5]” seems to be misplaced.

**ANSWER**: Corrected. Ref “[5]” moved to the end of the sentence.

Line 50: It should read predator***-prey*** interaction. Please revise.

**ANSWER**: Corrected.

Line 54: Please omit “in the community” because it suggests that you have modelled many more species.

**ANSWER**: Deleted as suggested.

Line 60: Delete the “.” in front of the reference.

**ANSWER**: Corrected.

Line 60: It should read “According ***to***”. Please revise.

**ANSWER**: Corrected.

Lines 62-65: Reads very general. Can you be more specific about your findings?

**ANSWER**: This is an introduction to what has been studied. More specific results are presented in the results.

Lines 80-83: Please, delete this paragraph as it is totally redundant to the text provided in lines 68-76.

**ANSWER**: Corrected. Deleted as suggested.

Lines 103: Please add “in the absence of the other species”.

**ANSWER**: Rephrased for other reasons.

Line 107: The total number of prey individuals harvested is given by a*C****S*** and not a*C!

**ANSWER**: Corrected.

Line 119: Please delete “of the” form the sentence as it is written down twice.

**ANSWER**: The lines 117-124 have been deleted.

Lines 122/123: This sentence reads odd. Please clarify.

**ANSWER**: The lines 117-124 have been deleted.

Lines 207/208: Please delete this sentence as it is part of the methods.

**ANSWER**: The sentence is deleted.

Lines 219, 221, 223, 225 & others: Why is the intrinsic growth rate called an environmental growth rate? Please clarify or revise.

**ANSWER**: The term “environmental” is deleted.

Line 219: What do you mean with an opportunistic prey? Please clarify.

**ANSWER**: The word ”opportunistic” deleted as unnecissary.

Line 244-246: This sentence does not add any explanation but restates the observation twice. Please revise.

**ANSWER**: Figure legend has been shortened and this observation has been removed from there.

Line 252/253: What do you mean with the order of the population sizes?

**ANSWER**: Corrected. We now use ”the relative population sizes”.

Line 254: Delete “to” from the sentence.

**ANSWER**: Corrected

Line 261: Please add a “to” in front of “a”.

**ANSWER**: Corrected.

Lines 273/274: Please delete Fig.3 in front of the subheading.

**ANSWER**: Lines 273-274 are not a subheading but the title of Figure 3 (according ro the

author instructions).

**Reviewer 2**

Reviewer #2: While the topic of the manuscript on a food web with the possibility of reversesd prey-predator roles is quite interesting, and could as such be relevant to a number of empirical systems, I think it is not yet matured enough to be ready for publication. First of all I think that the explanation of the food web, the role of the bacteria as prey as well as infectious disease should be more clearly explained using established nomenclature. Specifically the model should not only be described as a Lotka-Volterra predator-prey model, but as a combination of a standard Lotka-Volterra predator prey and a SIR model (an established approach to model infectious disease dynamics). There is a lot of confusion on terms throughout the manuscript, see the more detailed comments directly to the article in the attached pdf.

While the explanation on your parameters seems as such to be quite detailed, they are not at all clear. Specifically it is not clear to me what is the scientific basis that support the terms used, based on wet or dry-weight, to derive specific rates like assimilation efficiencies (L134-137). Also the units used are missing a relation to volume or area. The "attack rate" of the cucumber for bacteria to me would be better described as a 'filtration rate' and not an 'attack rate', where cucumbers filter a certain volume of sediment per unit time and therefore feed on bacteria proportional to the density of the bacteria in the sediment.

Why did the authors choose a model with state variables measured in terms of individuals instead of biomass? Since the body size of cucumber versus bacteria are several orders of magnitude apart from each other, I would find it more reasonable and easier to judge on the parameterization, if state variables and parameters would be indicated on a per unit biomass basis.

The result section is quite lengthy, but still not always on the point (see detailed comments in the pdf).
The authors could more clearly relate the analytical findings to the numerical results and specifically use the analytic findings to derive some general insight on the influence of certain parameters on coexistence and dominance patterns of prey and predator. These theoretical insights should then in the discussion be related to their relevance and implications for real systems, the specific study system, as well as other systems.

The discussion is not very well developed. The authors do neither highlight the main results nor put them adequately into context of existing work. The relevance of findings from this model for other model systems is very vague and it is not clear how and why the mentioned empirical systems for example the pike and stickleback system, or agriculture could be related to the investigated model system.

**Answer**: *Thank you very much your thorough, numerous and helpful comments. We have attempted to answer to all the comments above and those in the PDF file.*

Summary of comments (with answers) on PONE-D-21-07782_reviewer 2

**Page: 8**

**Page: 8**

Type: Highlight

It would be good if you could give examples right away. What cases does this refer to? Often such reversals come from different life stages etc. so it would be nice to either provide the full picture and/or to make clear what scenario the authors have in mind.

Answer: We have included a new clarifying section in the beginning of the introduction

**Page: 9**

**Page: 9**

Type: Highlight

sounds strange. As such it is its efficiency in infecting and converting predator biomass into own biomass, right?

Answer:We have reworded the text as suggested.

**Page: 9**

Type: Highlight

so the cucumber also digests the bacteria? Or do they just inhabit its gut, similar to human gut microbiomes?

The distinction is necessary in my oppinion. If the cucumber does not really feed (cannot exist on a diet of solely bacteria) it is more an example of disease-dynamics.

ASNSWER: the cucumber also digests the bacteria. We have revised the introduction explaining the problem more generally.

**Page: 9**

Type: Highlight

Please be more concrete of what this means. Can a cucumber exist on a bacteria diet alone? Or is taking up bacteria more a side effect of its feeding strategy, but not (a main) food source?

Not sure one can say that bacteria consume their host when infecting it. At least normally a different terminology is used, however, I can see the point of why using the same word. Still I think overall already here in the introduction the process of how the bacterium "consumes" the cucumber should be explained more clearly.

It should also be noted that the consumption rates are quite different between a (rather large) cucumber consuming many bacteria, (and probably needing a lot of bacteria for growth), while the bacteria, if infective, will multiply a lot, generating a large population which then may finally lead to the death of the cucumber through ...

ANSWER: The introduction is completely rewritten to improve the presentation

**Page: 9**

Type: Highlight

Corrected: parameterize

**Page: 10**

**Page: 10**

Type: Highlight

I think this statement is inappropriate here in the introduction. Something like this can be stated (if adequat at all) in the discussion.

ANSWER: Sentence deleted. Topic removed to discussion.

**Page: 10**

Type: StrikeOut

Answer: Deleted as suggested.

**Page: 10**

Type: Caret

system dynamics dependent on ... as well as

Answer: Corrected

**Page: 10**

Type: Caret

Results show that

Answer: Rewritten as suggested.

**Page: 10**

Type: Caret

critically

Answer: corrected as suggested-

**Page: 10**

Type: Highlight

I do not think that attack rate is the right word. As you describe the cucumber feeding mode further up, it sounds like it takes up loose sediment, but does not actively hunt for bacteria. Filtration/consumption rate might therefore be a more appropriate term.

ANSWER: Corrected as "grazing rate"

**Page: 10**

Type: StrikeOut

assume

ANSWER: The sentence has been deleled while rewritng the model section.

**Page: 10**

Type: Highlight

did you investigate this case? What parameter has to be set to zero to derive this cases? please be more specific.

ANSWER: If the the growth rates r_C or r_S is equal to 0, then the species can be considered as specialists.

Some examples of these have been shown in "Results" section as a point of comparison.

**Page: 10**

Type: Highlight

The predator has two states S and I, please explain that right away.

ANSWER: Now indecated in teh 1st sentence of model section.

As such your model is a mix of a S-I model used to model infectious disease dynamics, and a predator-prey model.

ANSWER: We challenge this interpretation. We have added argumetns in teh Discussion.

**Page: 10**

Type: Highlight

I assume it does all of that at the same time, according to different rates. So please rephrase accordingly. Also some of the rates will also change the S and C pop. So please also explain for them.

ANSWER: We have added more information on this in the new text in the third section in chapter "Modelling partial role reversal..."

**Page: 10**

Type: Highlight

No comments.

ANSWER: This is the first line of figure text

**Page: 11**

**Page: 11**

Type: Caret

at a constant infection rate

ANSWER: Corrected in the new figure caption

**Page: 11**

Type: Highlight

is this part of the figure caption? Otherwise it seems repetitive to the previous paragraph.

ANSWER: Yes, but the figure text has been rewritten.

**Page: 11**

Type: Highlight

distinctive from what? There are many models that also assume alternative resources/constant inputs

ANSWER: Corrected as "significant"

**Page: 11**

Type: Highlight

To be honest, I do not know why you separated the conceptual model from the description of the theoretical model. In my oppinion both can be combined. Then it would sound less repetitive and you can explain the concept which is directly linked to your equation system.

ANSWER: Corrected. We reorganized teh presentatin so that a general description of the system (with the figure 1) is presented before the model description.

**Page: 12**

**Page: 12**

Type: StrikeOut

by

ANSWER: Corrected

**Page: 12**

Type: Caret

terms

ANSWER: Corrected

**Page: 12**

Type: Highlight

I would say ri are growth rates and Ki the carrying capacities of prey and predator, respectively.

ANSWER: Corrected as suggested.

**Page: 12**

Type: Caret

for prey C

ASWER: Corrected as suggested.

**Page: 12**

Type: Highlight

again I think attack rate is not the right name and the units it is measured should fit to the actual feeding mode of a cucumber.

ANSWER: Corrected as "grazing rate"

**Page: 12**

Type: Highlight

it is not modelled as a type I functional response, since this would require to level off at above some critical prey density. What you used is a linear food uptake term.

ANSWER: We removed the statement about functional response.

**Page: 12**

Type: Highlight

should also be alpha, but seems to be an a

ANSWER: It is an a. However, due to the difficulties in telling apart alfa and italics a we changed the notation such that grazing rate (forrmer attack rate) is now denoted by "g"..

**Page: 12**

Type: Highlight

yes, this should be appropriate for the cucumber

ANSWER: No comment

**Page: 12**

Type: Highlight

I cannot see this point. If the cucumber just "ingests" sediment at a certain rate, then it would consume the bacteria in this sediment according to its relative abundance.

ANSWER: We maintain this interpretation. No changes done.

**Page: 12**

Type: Caret

the fraction of what? I guess the fraction that in fact is infectious/leads to an infection of the predator/host.

ANSWER: Correct! No changes made.

**Page: 12**

Type: StrikeOut

susceptible (please stick to your nomenclatur)

ANSWER: Corrected.

**Page: 12**

Type: StrikeOut

population

**Page: 12**

Type: StrikeOut

for me this would be an infection rate. I know that mathematically there is an equivalency, but biologically I would call it infection rate.

**Page: 12**

Type: Highlight

rather combine with previous sentence, e.g.

infects the predator pop at infection rate eSI, converting a corresponding fraction of the susceptable predator population to infected.

ANSWER: Corrrected.

**Page: 12**

Type: Highlight

lake stated previously, I think it is strange to first name different parameters and then refer to their meaning in the text in this sequential manner. I would rather name one followed by the explanation, name the second followed by the explanation ...

ANSWER: Corrected.

**Page: 12**

Type: Highlight

why µI? it seems to be also used for the susceptible predator.

Answer: Corrected. We changed the notation and denote the predator bacckground mortality or natural mortalisty as µ_S.

**Page: 12**

Type: Highlight

it is often called background mortality

ANSWER: No changes.

**Page: 12**

Type: StrikeOut

ANSWER: The whole section is removed

**Page: 12**

Type: Highlight

The model is a mix of a classic Lotka-Volterra predator prey and a S-I-R model.

ANSWER: The whole section is removed

**Page: 12**

Type: StrikeOut

I think these details are not necessary.

Please do not use a term like healthy harvest. If you can say the food uptake term of the susceptable predator is assumed to follow ....

ANSWER: The whole section is removed

**Page: 12**

Type: Highlight

I would not be too specific in the comparison to Lotka Volterra. It has been used and further developed in many aspects. So I think it is enought to state that it is a comb of Lotka-Volterra and a SIR model (see previous comment) and then just state some additional assumptions, like alternative resources ....

ANSWER: The whole section is removed and some discussion is presented in the Discussion.

**Page: 13**

**Page: 13**

Type: Highlight

suitable values for what? I guess for your study system, so please state so

Answer: Corrected as "values available in the literature".

**Page: 13**

Type: Highlight

what is ecological efficiency?

Answer: Corrected. We now use assimilation efficiency in our calculations.

References are given.

Type: Text

please use the same abbreviations for parameters as introduced in the previous section.

**Page: 13**

Type: Caret

another bacterium (of similar size)

Answer: Corrected as suggested.

E.coli and V.splendidus are approximately of similar size. To be precise, Bacteria of genus Vibrionacea are often a bit larger than E.coli, but the difference in mass, or digestible matter, is not overwhelming. Also the exact assimilation efficiency for V.splendidus is still under research, and therefore we have used a very conservative estimate, which lessens the impact of size difference

**Page: 13**

Type: Caret

the bacterium

Answer: corrected as suggested by the referee

**Page: 13**

Type: StrikeOut

Answer: Corrected

**Page: 13**

Type: Caret

Year or number missing

Answer: Corrected!

**Page: 13**

Type: Highlight

How, why can we calculate converssion efficiencies from dry/wet weights?

As such one would need studies that looked at how much consumed prey biomass led to how much growth in predator biomass.

This here would just reflect the predator to prey size ratio.

Answer: We are using assimilation efficiency, which is usually measured as the ratio of food assimilated to food ingested, A=[(C-E)/C]*100, where C is consumed energy, and E is egestion, or discharge of unused material. In effect, A*C=C-E=P+R is the increase in mass, either by growth (P) or reproduction (R). C and E are measured as dry mass. Assimilation efficiency is similar to energy budget calculations. (Valiela I. Marine Ecological Processes, 3ed. 2015; Springer) This is also very similar to the carbon budget experiments that have been done specifically with A.japonicus (e.g. Yang et al. 2018).

**Page: 13**

Type: Highlight

Again, I find it strange to calculate feeding range from observed population densities. This can at best be a very rough estimate. Are there no studies that directly investigated how much volume of sediment a cucumber can take up per hour or per day?

Answer: Because the sea cucmber can be considered a grazer, the available food volume depends on the sea cucmber density. We are ausing the measured sediment ingestion rate in calculating the grazing rate g. In formula g=m_{Aj}*f/(ρV) the parameter f is the volume of sediment eaten per hour (Sun et al. 2015).

**Page: 13**

Type: Highlight

Empty comment. No reaction.

**Page: 13**

Type: Highlight

Overall the explanations are a bit bulky and it is not always clear why certain functional terms were used to derive specific parameters. If it is based on available literature please state so and add more explanations.

'Answer: We haved attempted to repost the parametrization more carfully. Most to the related text is rewrtitten.

**Page: 14**

**Page: 14**

Type: Highlight

Is any of this related to AKS?

This here, if reported as such in the literature makes sense to me for a sediment filtration rate.

Answer: If AKS means K_S, then it is related. In a way this is sediment filtration, but the mechanical grazing activity is actually food item picking using the feeding tentacles.

**Page: 14**

Type: Highlight

the nominal portion?

It is as such a filtration rate, which will then be proportional to the food uptake of prey, dependent on the prey density in the sediment.

Answer: "nominal" deleted

**Page: 14**

Type: Highlight

I dought that the cucumber selects for bacteria in the sediment.

You can rather motivate the observation of values around this basic value because of uncertainties on how the value was estimated or due to natural/individual variations in filtration rates.

Answer: According to Plotieau et al. (2013) holothurians seem to specifically select bacteria from the sediment. Also Song et al. (2018) found out that A.japonicus selects food accoring to preferences, although they did not focus on bacteria. Theoretically selection would be good choice, because bacteria are much better digested than plant detritus (algae, eelgrass) or ash containing fecal detritus.

**Page: 14**

Type: Highlight

I guess this could only be veryfied if the desnities of bacteria/cucumber you derive lie within realistic ranges observed in the natural environment. Is this the case?

Were your results dependent on assumptions on KC and KS?

Answer: Both the sea cucumber and bacteria population densities have been taken from research that measured population densities in nature. To simulate the natural ratio of population densities, we calculate KC for the area that corresponds to the area needed for sea cucumber at KS. Hence we use the population density estimations for both species. The exact numerical results depend on the KC and KS, but the general results stand even when KC changes to unnatural densities, increasing or decreasing by 100 or 1000. Sea cucumber KS affects only the magnitude of the results.

We tested different values of the carrying capacity of the sea cucumber but did not find any new results to report.

**Page: 14**

Type: Highlight

The units completely miss the area relationship.

It should be ind/L or ind/m² or something like this, similar to the food uptake rate/filtration rate of the predator for its prey which should have some relation to volume or area that can be filtered per unit time.

Answer: We have incorporated the areas/volumes in the model parameters to simplify the notation. Because the model uses grazing rate that is the fraction of sediment eaten from the total volume of available sediment (and prey), we can embed the volume within the grazing rate

**Page: 15**

**Page: 15**

Type: Highlight

this is not the typical way to suggest an interval. please use mathematically appropriate bracketing.

Answer: Corrrected

**Page: 15**

Type: Text

In my opinion it is unnecesary to print all equations (4-6). It is enough to state that Eq. 1-3 were assumed to equal zero.

ANSWER: Corrected

**Page: 15**

Type: Highlight

is there a minus missing in front of the 13? 10^[-13] ? Otherwise the difference between eCS and eIC seems to be unrealistically high.

Anyway the values seem strange to me. Why should the conv eff of prey (bacteri) to predator (cucumber) be less then from pred to prey? Measuring it at the basis of individuals makes it a bit hard (in my oppinion) to judge on these numbers, since the biomass of a bacteria versus a cucumber is several orders of magnitude apart from each other.

Answer: Exactly. The biomass of a bacteria is several orders of magnitude less than that of the sea cucumber. Thus, eating one prey (bacterium) does noty provide much energy to tjhe sea cucumber. On contrary, one sea cucmber is worth of several bacteria in energetic terms.

**Page: 16**

**Page: 16**

Type: StrikeOut

ANSWER: DELETED

**Page: 16**

Type: StrikeOut

Solving Eq. 6 for I and replacing it correspondignly in Eq. 4 and 5, ...

**Page: 16**

Type: Highlight

**Page: 16**

Type: Highlight

**Page: 16**

Type: Highlight

I guess you solved Eq 6 for I and then replaced the expression by z? Otherwise one would not end up with eq. 7 and 8.

Answer: Corrected. Rewritten.

**Page: 16**

Type: Highlight

This information should be given before you state Eq. 7 and 8.

ANSWER: Corrected

**Page: 17**

**Page: 17**

Type: StrikeOut

ANSWER: Corrected

**Page: 17**

Type: Highlight

How did that depend on parameters? What was changed to derive one or the other outcome? Please specify. Maybe some general conditions can be derived from the analytic equilibrium solutions?

Answer: Not valid any more after rewriting the section.

**Page: 17**

Type: Highlight

what does that mean?

ANSWER: Not valid anymore after rewriting the section.

**Page: 17**

Type: Highlight

**Page: 18**

**Page: 18**

Type: Caret

the

ANSWER: Not valid any more.

**Page: 18**

Type: Highlight

What about the influence of carrying capacities which could differ in different habitats?

Did you find a stable equilibrium?

ANSWER: Not valid any more after rewriting the section.

**Page: 18**

Type: Highlight

It is a bit confusing, this section reads in part like a result section, but in fact it is still the methods, so please just describe here how you did your analysis, what paramters were varied in which way, what step sizes? Was it distinct combinations or a continuation etc.... How many time steps did you follow the dynamics ...

ANSWER: The section has rewritten such that all the results have been removed from here.

**Page: 18**

Type: Highlight

not crucial for what?

ANSWER: Corrected :"does not crucially affect the prey population size ".

**Page: 18**

Type: Caret

rc

ANSWER; Corrected

**Page: 18**

Type: Highlight

I would not call it outside growth rate. There is the growth rate rC and there is the infection rate eSI.

ANSWER: Corrected

**Page: 18**

Type: Highlight

shall this be sub-headings, or would you place the figures here? This is unclear to me. If a subheading, then please without the reference to the figure. This should be given in the text.

**Page: 19**

**Page: 19**

Type: Caret

represent

Answer: Corrected

Type: StrikeOut

**Page: 19**

Type: Caret

in Fig. 2 are

ANSWER: This is figure caption for Fig.2

**Page: 19**

Type: Highlight

This is already indicated in the table. Please do only explictely name numbers and ranges if they differ from the default.

ANSWER: Corrected

**Page: 20**

**Page: 20**

Type: Highlight

word missing? doesn't sound correct.

ANSWER: CORRECTED

**Page: 20**

Type: Highlight

would probably be good to show this already in the analytic solution section, or to refer here back to the respective expression.

What happens if a21 = 0? as mentioned in the next sentence?

Answer: Corrected. An analysis of the turning point equilibria is added to the end of chapter "population equilibria" (eqs (11-12)). In addition,

**Page: 20**

Type: Highlight

sound strange, try to rephrase

ANSWER: REWRITTEN

**Page: 20**

Type: Highlight

ANSWER: Corrected as " A strongly ..."

**Page: 21**

**Page: 21**

Type: Highlight

This is an interesting aspect. What are the thresholds and how are they related to the relative differences in growth rate between predator and prey and differences in the carrying capacities?

ANSWER: Rewritten.

**Page: 21**

Type: Highlight

How is that informative on what? I do not see why this would matter? Or is one a zoomed in figure of another one?

Also the labelling is missing from the figures at the end of the manuscript.

ANSWER: The sentence has been deleted.

Figure 3 has been revised.

**Page: 21**

Type: Highlight

fits to results from classic Lotka- Volterra predator-prey interactions (food chains)

ANSWER: NOT CLEAR I ANY REACTION ASKED.

**Page: 21**

Type: Highlight

I would rather use words as high or low instead of weak and strong. Not only here but throughout the manuscript.

ANSWER: Corrected

**Page: 22**

**Page: 22**

Type: Caret

settle on a

ANSWER: Corrected

Type: StrikeOut

**Page: 22**

Type: StrikeOut

ANSWER: See the next comment

**Page: 22**

Type: Caret

settle on a

ANSWER: Corrected as suggested.

**Page: 22**

Type: Highlight

ANSWER: Corrected

**Page: 23**

**Page: 23**

Type: Highlight

From this assumption it follows that the growth rate must be larger than the mortality rate µI, you should describe this.

ANSWER: The revised wording is now "eigenvalue ... is negative."

r_S>>mu_I so it can be easily checked.

**Page: 23**

Type: StrikeOut

ANSWER: Corrected

**Page: 25**

**Page: 25**

Type: Highlight

repetitive with previous sentence.

ANSWER: Sentence removed.

Say a bit more about what parameters are critical to fulfill the conditions. What are the most influential once, try to motivate what the equations tell us with respect to the biological rates.

ANSWER: The possible extinction of the predator is now commneted just after the analysis.

**Page: 25**

Type: Highlight

I would use different wording, see previous comments also on model type. For me the prey (bacteria) can infect the predator, I would not call it an attack.

ANSWER: Sentence corrected as asked.

**Page: 25**

Type: StrikeOut

ANSWER: Corrected.

**Page: 25**

Type: Highlight

A

ANSWER: Comment unclear.

**Page: 26**

**Page: 26**

Type: Caret

both have alternative resources/ can grow in the absence of their respective prey or host.

ANSWER: Rewritten as suggested.

Type: StrikeOut

**Page: 26**

Type: Highlight

I would not call it like this. As such they have alternative resources, OK, but I would not even mention that they are therefore generalists, especially since you only consider the direct interaction with one prey, one predator/host

ANSWER: The sentence has been removed.

**Page: 26**

Type: Highlight

This is largely repetitive with the introduction/methods section. Please highlight more the main results. The description on the main aspects of your food web that sets it apart from other food webs should be brief, avoiding to much repetition.

ANSWER: This comment on foodwebs was removed from the Introduction and shortened here as well.

**Page: 26**

Type: Highlight

What does this mean? Either it is very trivial, or there is a meaning I do not get?

ANSWER: The sentence is not exactly true so it was deleted.

**Page: 26**

Type: Highlight

please rephrase, this sentence is not understandible

ANSWER: Corrected.

**Page: 26**

Type: Caret

It has to be made more clear that there is a certain range of infectivity where the predtor profits from the increasing prey availability, until infectivity gets so high that the predator population decreases and finally goes extinct.

This feedback mechanisms remindes me of dynamics that can also happen in food webs with stage-structured populations.

It is definitely interesting (and should therefore be highlighted) that there is a hump shaped relationship and some intermediate range of infectivity (or other parameters) for which the predator (or both prey and predator) profit.

ANSWER: Lines 390 - 397 are deleted.

Type: StrikeOut

**Page: 26**

Type: Highlight

where is this shown?

Answer: Lines 398-401 deleted.

**Page: 26**

Type: Highlight

you have added a logistic growth term to your equations, so what do you mean?

ANSWER: Lines 398-401 deleted

**Page: 26**

Type: Highlight

What do you mean with extensive simulations? You only varied some parameters, not others. You did not test different carrying capacities. I would guess that there exist regions where the system would exhibit oscillations.

ANSWER: The four lines 398-401 have been deleted.

**Page: 26**

Type: Highlight

This sentence is unclear. Please try to rephrase. The predator does not become extinct if the the prey is infective as such. Only if infectivity gets too high, right?

ANSWER: Rewritten as "if the prey population is infective enough"

**Page: 27**

**Page: 27**

Type: Highlight

How does this example relate to your model? If you want to relate this study to yours you have to give more information.

ANSWER: We agree with the referee that these ideas are far-fetched. and immature We removed lines 413-419.

**Page: 27**

Type: Caret

in what examples? The one example [3] ?

ANSWER: As above, lines 413-419.

Type: StrikeOut

**Page: 27**

Type: Highlight

totally unclear to me what is meant and how it relates to the presented study.

ANSWER: ANSWER: As above, lines 413-419.

**Page: 27**

Type: Highlight

?

ANSWER: ANSWER: As above, lines 413-419.

**Page: 27**

Type: Caret

d

ANSWER: Corrected. But not valid any more.

Type: StrikeOut

**Page: 27**

Type: Highlight

do you have organisms in mind for which this could be relevant?

ANSWER: Lines 427-429 deleted.

**Page: 27**

Type: Caret

r

ANSWER: Corrected

Type: StrikeOut

**Page: 27**

Type: Highlight

NO COMMENT?

**Page: 28**

**Page: 28**

Type: Highlight

This would be similar to increasing the mortality rate on the prey in your study system. From your equations you can tell if that would have the envisioned effect.

This section seems a bit too sepcific on possible experiments.

ANSWER: Lines 430-437 deleted.
