# Supplementary material for: Infective prey leads to a partial role reversal in a predator-prey interaction
Source: PLoS One. 2021 Sep 17;16(9):e0249156. doi: 10.1371/journal.pone.0249156 (PMC8448379; doi:10.1371/journal.pone.0249156)
Supplement: Attachment — Submitted filename: Response to Reviewers.docx [file pone.0249156.s004.docx]

**Response to the Academic Editor and the Reviewer**

**Academic Editor**

… I would like to suggest three potential ways to mediate the issue: (1) provide a better connection with the existing literature in infectious disease models in general, (2) compare the model and results with the Nicholson-Bailey model, or (3) make the review comments available to the public. The originality of the research is one of the conditions for publication in the PLOS One, and the readers will need to know how the new knowledge is built on the existing ones. …

**Author Responses to the Academic Editor**

Thank you very much for the second round of the referee process.

We really appreciate the comments by the Academic Editor and the third reviewer. We have revised the MS following the advices of the Academic Editor and the the reviewer.

In particular, we now provide a better connection with the existing literature in infectious disease models in general (Option (1) provided by the Editor). We introduce the well-established disease models preliminary in the introduction. A more detailed and lengthy comparison of the models is presented in the Discussion, where we characterize the disease model classes and point out crucial differences between them and our model.

Addressing Option (1) also covers a comparison with the Nicholson-Bailey model as we have compared our model with the four most well-known disease model classes, one of which is the host-parasitoid model, also known as Nicholson-Bailey model (Option (2) provided by the Editor).

Thus, we conclude that that there is always at least one crucial difference between our model and any of the other well-known disease models. Thus, we continue defend our argument about role reversal in a predator-prey interaction.

As an important part of our revision we improved our argument about the *Vibrio splendidus* being significant in the bacterial community and in the diet of the *A japonicas* (as was requested by reviewer 3).

If the current version of the MS cannot be accepted for publications in the PLOS One, we would be happy to follow the third option (3) where the review comments will be made available to the public.

6. **Review Comments to the Author**

Reviewer #3: Review for: Infective prey leads to a partial role reversal in a predator-prey interaction

(**Bold font below is due to the authors of the MS**.)

The science (methods and results) appear sound, however, as a draft, **lack of context** or **awareness of large bodies of important work** is disconcerting, and as a consequence the science doesn't add much to our knowledge of ecology.

The authors implement a straightforward Lokta-Voltera style coupled differential equation system to model predator-prey dynamics, though not grounded in any data, and describing this system as a predator-prey system seems a bit contrived. The results are unsurprising: when a "prey" species begins to behave increasingly like a pathogen, then the "predator" (it's hard for me to call a bacteriophage a predator) tends to die and the pathogen does better, while when a "prey" is less and less pathogenic (is more and more tasty), the predator does better.

Although I think the science is basically sound (though not particularly consequential), I have some issues with the way the authors have cast the system. The idea that this is a special case of a predator-prey system is a big stretch, and I feel the system is probably **much better characterized as a special case of a host-pathogen system**, for which there has been a great deal of previous thought, research, and modelling work to build upon and contextualize the system with. Although I was eventually convinced that could think of V. splendidus as a consequential food source, I had to do a lot of digging in the literature -- the authors **did not cite any papers the indicated if the biomass of V. splendidus was significant in the bacterial community** -- **something they need to set up the model as they have**. Instead, I had to convince myself that V. splendidus would form a significant fraction of the bacterial biomass in this or similar systems by reading other papers on the topic. If V. splendidus is never or rarely of significant biomass, the system *definitely* has to be considered host-pathogen problem and not a predator-prey problem. So **evidence that V. splendidius is a large fraction of the bacterial biomass in some relevant circumstances needs to be provided in the introduction to set up the problem**, otherwise the model simply isn't well enough grounded in any real system to be helpful or informative at all.

I think **more problematic is the lack of recognition, citation, or development of the model presented's relationship to two other key bodies work**. The first is the enormous body of epidemiological and host-pathogen models, which are of course highly informative of the dynamics modeled here. At least as important an oversight is the classic but uncited Nicholson-Bailey model (Nicholson, A.J. and Bailey, V.A., 1935. The Balance of Animal Populations), who built a Lokta-Voltera predator-parasitoid model very, very similar to the model presented in this paper (and did so 85 years ago), and the large body of work, papers, and model extensions that grew from that classic model. None of that body of work is cited here, and **it seems the authors are unaware of it**. This is a problem given its similarity in both form and substance. So **that context needs to be added to both the intro and discussion**.

Currently the discussion, which is normally the place where authors place their findings into the context of the larger body of scientific work, has no references (which is a-typical to say the least) and mostly serves to reiterated the results. That's just not acceptable.

Finally, the authors have made minimal effort in terms of labels, colors, font sizes, line width, legend, etc. with respect to making their **figures** intuitive and informative.

Altogether this paper just feels sloppy, it doesn't seem to be aware of other important and relevant bodies of work and is consequently reinventing the wheel to some degree with a contorted pitch about predator-prey role reversals. It's really a host-parasite or host-pathogen problem, which have been much better worked. If this were any other journal, I would not recommend publication. Here I can only ask that at the very least **the authors work to better cite and ground their work in relevant literature and rework their figures** so they are of reasonable quality and readability.

**Author Responses to the Reviewer**

Thank you very much for the critical and competent evaluation of our MS. We agree with several issues and points raised by the reviewer in his/her report.

We made extensive revisions to improve the MS (following the justified advices of the reviewer), and also, to defend our view on the problem.

**First**, the reviewer states that the “**evidence that V. splendidus is a large fraction of the bacterial biomass in some relevant circumstances needs to be provided in the introduction to set up the problem”.**

This is an important point in order to better pose the problem. In response, we have added into the Introduction the following text on the role *V. splendidus* in the diet of the A. japonicus (supported with new adequate references, lines 62-68): *V. splendidus* is an efficient decomposer, which allows it to thrive in many environments [21,22]. To this end, *Vibrios* form a notable fraction of the bacterial flora in the sediment. Especially in nutrient rich areas near the range of *A. japonicus* *Vibrios* form one of the most abundant bacterial groups [22,23]. Bacteria are most abundant in the detritus, where the bacterial density can be hundredfold compared to the sea water above the detritus [20,23].

We also comment this in the Discussion (lines 480-485): Decomposer bacteria are abundant in the sediment [20,22,23], especially at the nutrient rich sea grass beds [23]. Because sea grass beds and the adjancent areas host also large numbers of detrivores and bacterivores that include sea cucumbers [23], bacteria play important part in the food web [11,18], although the details of the interactions are still poorly understood.

Thus, we continue defending our view that bacteria form an important food source for the sea cucumber, and that *A. japonicus* can be treated as a predator to *V. splendidus*.

**Second**, we agree that a proper comparison of our model to several well stablished disease models was lacking in our previous version of the MS. Even if our focus was in the predator-prey interaction we now feel and agree that this comparison is most important to communicate to the readers. We have now raised this issue in the introduction on a general level (lines 94-104).

We continue comparing the similarities and differences between our model and other disease models in more detail in the Discussion (lines 521-571). In our lengthy and detailed comparison we argue that there is always at least one crucial difference between our model and any of the well-known disease models. We are not aware of any source reporting a theory for a similar species interaction with our case.

Thus, we continue to consider or study be an example of role reversal interaction in a predator-prey interaction, even if the prey happens to be a parasite.

Third, we have redrawn Figures 2-6 and the figure texts in order to improve their readability and clarity.
